# Metabonaut: an open educational resource for learning reproducible, script-based untargeted LC-MS/MS metabolomics

**DOI:** 10.1007/s11306-026-02538-x

**Published:** 2026-10-03

**Authors:** Philippine Louail, Marilyn De Graeve, Alexandru Mahmoud, Daniel Marques de Sá e Silva, Kozo Nishida, Vilhelm Suksi, Anna Tagliaferri, Gabriele Tomè, Vinicius Verri Hernandes, Johannes Rainer

**Affiliations:** 1 Institute for Biomedicine, Eurac Research, Via Volta 21, 39100 Bolzano, Italy; 2 Chair for Bioinformatics, Faculty of Mathematics and Computer Science, Friedrich Schiller University Jena, Jena, Germany; 3 Limerick Digital Cancer Research Centre, Health Research Institute, School of Medicine, University of Limerick, Limerick, Ireland; 4 Department of Chemistry, Aristotle University of Thessaloniki, Thessaloniki, Greece; 5 Biomic AUTh, Center for Interdisciplinary Research and Innovation (CIRI-AUTH), Balkan Center, Thessaloniki, Greece; 6 Laboratory for Biologically Inspired Computing, RIKEN Center for Biosystems Dynamics Research, Kobe, 650-0047 Hyogo Japan; 7 Department of Computing, University of Turku, Turku, Finland; 8 Faculty of Agricultural, Environmental and Food Sciences, Free University of Bozen-Bolzano, Bolzano, Italy; 9 Sensing Technologies Laboratory (STL), Faculty of Engineering, Free University of Bozen- Bolzano, Bolzano, Italy; 10 Department of Food Chemistry and Toxicology, University of Vienna, Vienna, Austria

**Keywords:** Untargeted metabolomics, Reproducible research, FAIR principles, Literate programming, *Metabonaut*, Open educational resource

## Abstract

**Background:**

Two decades after the Metabolomics Standards Initiative, public deposition of raw LC-MS/MS data into archives such as MetaboLights, Metabolomics Workbench, GNPS and MassBank has matured, but the analyses that turn raw files into reported feature tables remain difficult to share, audit and reuse. The FAIR principles, originally formulated for data, later extended to research software and to workflows, make a strong case that this gap matters; we adopt that view and ask how an educational resource can help researchers cross it.

**Aim of review:**

This is an educational review where we describe the principles of FAIR and reproducible analysis as they apply to untargeted LC-MS metabolomics, and we show how those principles are realised in *Metabonaut*, an open educational resource built on top of the R for Mass Spectrometry/Bioconductor ecosystem. *Metabonaut* provides executable example workflows from raw mzML files to annotated feature tables, demonstrates interoperability across programming languages, and is built with and for the community.

**Key scientific concepts of review:**

After a brief tour of the underlying software ecosystem and of literate programming, the review discusses three topics, each through one or more *Metabonaut* vignettes: visual inspection and quality control, reuse of public archives at scale, and cross-language interoperability. Together, they teach how to carry FAIR practice from data to analysis: making the analysis itself, and not only the data it consumes, shareable, auditable and reusable. The source code of Metabonaut and links to pre-rendered workflow documents are available at https://github.com/RforMassSpectrometry/Metabonaut.

## Introduction

### The reproducibility gap in untargeted metabolomics data analysis

Two decades after the Metabolomics Standards Initiative (MSI) published its minimum-reporting recommendations (Fiehn, 2002; Sumner et al., 2007), the data side of the field has matured. MetaboLights (Yurekten et al., 2024) now hosts 3,080 studies, Metabolomics Workbench (Sud et al., 2016) 4,379 publicly available studies, and GNPS/MassIVE (Wang et al., 2016) spans 19,734 mass spectrometry datasets (all as of 6 July 2026). Counts alone, however, say little about reusability. An evaluation of 399 public studies across four metabolomics repositories found that none of the MSI biological-context reporting standards was met by every study, with compliance for individual standards ranging from 0 to 97% (Spicer et al., 2017). A study with poor metadata is difficult to reuse even when its raw files are open. Raw-data deposition (open data) is increasingly an expected part of publication, but the data analysis side (open analysis) has not kept pace. A reader who downloads a deposited set of mzML files can rarely recompute the feature table reported in the corresponding paper: parameter values are only partially documented, manual exclusion and filtering steps are described in prose if at all, and the metadata required to reproduce the downstream statistical analysis is often missing. The consequences of unreproducible data analysis are not hypothetical: in a well-documented case, undocumented, spreadsheet-based errors in a set of genomic predictors went undetected until a forensic re-analysis, by which point the flawed models had been used to assign patients in clinical trials (Baggerly & Coombes, 2009). Scripted, transparent analyses make such errors visible and correctable before they propagate.

This observation is not new. Stanstrup et al. (2019), in their broad survey of R-based metabolomics tooling, observed that the availability of open-source software does not in itself guarantee reproducible analyses, and that the gap between code being public and an analysis being re-executable is wide. Mendez et al. (2019) argued along similar lines that collaborative, open data-science practices (i.e. notebooks, shared environments, version control) are needed before deposition alone can deliver on its promise.

### FAIR analyses, not just FAIR data

The Findable, Accessible, Interoperable and Reusable principles (FAIR) were originally formulated for data (Wilkinson et al., 2016). They have since been extended to research software (Barker et al., 2022) and increasingly to workflows. We follow that extension here. We treat a data analysis as FAIR when its code, computational environment, narrative description and intermediate outputs are themselves findable (deposited under a persistent identifier), accessible (retrievable under clearly defined conditions, which may include authentication where a service requires it), interoperable (built on open standards and shared representations such as mzML, mzTab-M and HDF5), and reusable (documented, licensed and described well enough for others to adapt). Table 1 lists the practical artefacts that follow from each of those four properties for a typical untargeted LC-MS workflow. This is the conceptual frame for the remainder of the review.

**Table 1:** Practices that make an untargeted LC-MS metabolomics analysis FAIR (Wilkinson et al., 2016), with the corresponding open tools used in this review

FAIR property	What it means for an analysis	Practical artefact
Findable	The analysis has a persistent identifier and is discoverable alongside the data it produced.	DOI-minted code release; deposited workflow; cross-link to the raw-data accession.
Accessible	The code, environment and intermediate outputs can be retrieved under clearly defined conditions, which may include authentication.	Public repository; open-license container image; archived rendered notebook; documented access conditions where a service requires an account.
Interoperable	The analysis consumes and produces data in open, standard formats and runs across languages.	mzML/mzTab-M/HDF5 inputs and outputs; R↔Python and R↔Java bridges.
Reusable	The analysis is documented, licensed and structured so others can adapt it.	Literate Quarto vignettes with narrative + code + output; explicit licence; modular package functions.

These notions are related but not equivalent, and we use them in specific senses throughout. Open concerns whether resources can be freely accessed and reused. FAIR accessibility, by contrast, explicitly permits authentication and authorization where a resource requires it, so a resource can be FAIR without being anonymously open. Reproducibility concerns the ability to reconstruct or re-execute an analysis and obtain the expected result. Metabonaut aims at all three, but they can be satisfied independently: some routes we describe require an account (the Bioconductor Workshop platform) or user credentials (SIRIUS structure searches) and are therefore accessible under defined conditions rather than anonymously open, while remaining fully reproducible for a reader who holds those credentials.

### The training gap

Most analytical chemists and biologists entering metabolomics first learn data analysis through vendor software or tools with graphical interfaces, which lower the entry barrier to the field. Some graphical tools also support reproducibility directly: for example, mzmine can export a batch file that records and replays a complete processing sequence (Schmid et al., 2023). *MetaboAnalyst* likewise lets a user download the R command history of a web session and re-run it locally through its companion package (e.g. MetaboAnalystR; Chong & Xia, 2018). More generally, however, the artefacts a graphical session leaves behind are less amenable to line-by-line inspection, version control and reuse than a script, and for practitioners without a computational background the step from a graphical workflow to a fully scripted one remains substantial. This step is the main hurdle to adopting the practices described here, alongside an understandable reluctance to invest the time. In our experience training PhD students and researchers entering the field, a few focused weeks are enough to become productive, and the return, analyses that are transparent, reusable and re-executable, repays that investment. Further, scripts containing only data analysis commands but no explanations or comments are difficult to understand. Existing teaching resources have only partially closed this gap: they tend to be tool-centric (the *xcms* package vignettes) or platform-centric (the Galaxy Training Network, Workflow4Metabolomics) rather than workflow-centric, and few of them follow a single, realistic dataset from raw files to interpretable results. *Metabonaut* was built to address that gap. It is designed as a community resource: the workflows it contains are not specific to any one project, the contribution model is open, and the executable vignettes are intended to be read, run and adapted by anyone learning the R-based metabolomics stack.

We use *Metabonaut* as the worked example throughout this review, not as the only route to FAIR metabolomics analysis, but because it covers the full path from raw data to annotated feature tables and makes the underlying design choices inspectable. Two sections lay the groundwork: the first introduces the R for Mass Spectrometry ecosystem and the shared data structures its packages exchange, and the second presents literate programming, the medium in which prose, code and output are kept in a single executable document. A third section describes the design of *Metabonaut* and the pedagogical choices behind its vignettes. The three topics of this review then follow, one per section and in this order: visual inspection and quality control; reusing public data and scaling to large cohorts; and interoperability to extend analytical capacity.

### The worked dataset

Throughout this review we refer to two publicly deposited datasets that are used within *Metabonaut*. The first is a small LC-MS/MS data set from blood plasma samples of individuals with cardiovascular disease and healthy controls, deposited in MetaboLights as MTBLS8735; we will call it the *Metabonaut plasma example* and most worked examples in this review are based on it. Its use is strictly didactic: the subset is small and the pooled QC samples originate from a different sample collection than the study samples, with only four QCs available. These are deliberate teaching conditions rather than a biological study of cardiovascular disease, and no conclusion about the condition should be drawn from the comparison. We consider the imperfection instructive, since it lets learners see when a standard method may not perform reliably. One topic, analysis at the scale of thousands of files, requires a larger dataset; for that we use a second MetaboLights study, MTBLS93 (a population-scale, non-targeted serum metabolomics dataset from the Swedish TwinGene case-cohort), which we will call *the large-scale study* (Ganna et al., 2014). The *Metabonaut* workflows start from mzML files: conversion from the instrument vendor’s raw format to this open standard, with an open-source tool such as msconvert (Chambers et al., 2012) from the ProteoWizard suite, is assumed to have happened already and is not covered here.

## Bioconductor and the R for mass spectrometry ecosystem as a foundation

### Architecture

The Bioconductor project (Gentleman et al., 2004) provides free, open-source software for the rigorous and reproducible analysis of data from current and emerging biological assays. As part of Bioconductor, the R for Mass Spectrometry initiative (Rainer et al., 2022, 2025) adopts the same conventions, i.e., strict package review, shared base classes, regular release cycles, and applies them specifically to MS data analysis. The result is a software ecosystem in which each package does one thing and shares its data structures with the others.

At the lowest level, *Spectra* (Rainer et al., 2025) abstracts the representation of MS data independently of where they are stored, exposing the same R interface whether the data live in memory, in files on disk, in a database, or in a public repository. *Chromatograms* (Louail et al., 2026) plays the analogous role for chromatographic data. *MsExperiment* ties spectra, chromatograms and sample metadata into a single experiment-level container.

**Fig. 1:**
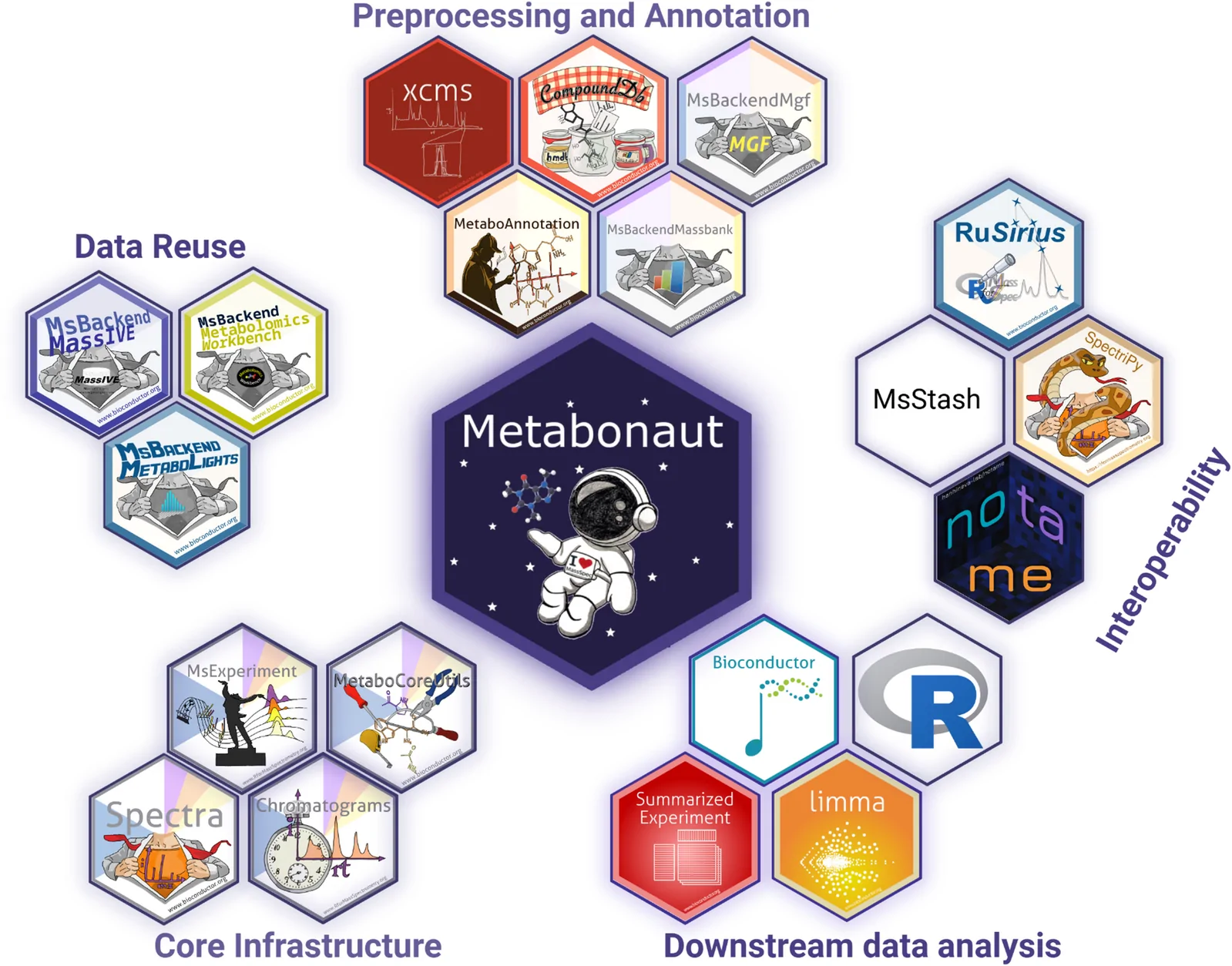
Metabonaut teaches how to use the open source ecosystem to produce FAIR analysis in metabolomics

Building on this foundation, *xcms* 4 (Louail et al., 2025), one of the main preprocessing tools in the field, has been refactored onto this infrastructure so that its results integrate natively with the rest of the ecosystem. *MetaboAnnotation* and *CompoundDb* (Rainer et al., 2022) expose a uniform annotation interface across in-house and public reference data, e.g., from MassBank (Horai et al., 2010), HMDB (Wishart et al., 2022) or GNPS. The *MsStash* suite of packages standardises import and export of MS analysis objects, and the *RmzTabM* package provides the mzTab-M (Hoffmann et al., 2019) import and export for *R* objects, allowing results to be both written to and read back from the standard format. *SpectriPy* (Graeve et al., 2025) bridges Bioconductor *Spectra* to Python’s *matchms* (Huber et al., 2020) and *spectrum_utils* (Bittremieux, 2020), while *RuSirius* interfaces RforMassSpectrometry to SIRIUS (Böcker et al., 2009; Dührkop et al., 2019) to bring formula and structural annotation into the same R session. Figure 1 shows how these packages connect; the same figure also serves as a mental map for the rest of the review.

### Why this matters pedagogically

Bioconductor’s commitment to shared data structures (the common classes such as *Spectra*, *MsExperiment* and *SummarizedExperiment* (Martin Morgan, 2017) that packages exchange) is not just a software-engineering choice; it is what makes the ecosystem teachable. A learner who masters one *Spectra* object, one *MsExperiment* object and the broader Bioconductor data model carries that mastery across preprocessing, annotation and statistics; the same objects flow through every step of a workflow without conversion glue, so the analyst can concentrate on what to do with the data rather than how to hold it. LC-MS data are not two-dimensional: intensity is recorded across both retention time and *m/z* so the data does not fit a flat table. A single study can also hold hundreds of thousands of spectra and tens of gigabytes, more than a session can keep in memory. A *Spectra* object keeps a tabular interface over that structure while leaving the data on disk or in an archive rather than loading it into memory, so the same code runs unchanged on three files or on several thousand.

That modularity is also what makes a coherent teaching resource possible in the first place. *Metabonaut* demonstrates ways to wrap the ecosystem; it does not re-implement it and each vignette can pick up where the previous one left off. The same property explains why the ecosystem extends so naturally to large-scale and cross-repository analyses: a new backend, whether for an on-disk HDF5 store or for a public archive, slots into the same *Spectra* abstraction without affecting any downstream code.

The R for Mass Spectrometry project is one of several open-source options for untargeted LC-MS metabolomics, each with a different focus: some target downstream statistics (e.g. *MetaboAnalystR*; Chong & Xia, 2018), clinical normalization and quality control (e.g. *notame*; Klåvus et al., 2020) or non-target screening (e.g. *patRoon*; Helmus et al., 2021), and others provide graphical, hosted workflows (e.g. Workflow4Metabolomics; Giacomoni et al., 2015), (mzmine; Schmid et al., 2023). These are complementary rather than competing choices, and they are often used together (Table 2*)*.

**Table 2:** Open-source environments for untargeted LC-MS metabolomics, organised by design centre

Tool	Language/interface	Design centre	Primary audience
Metabonaut/R for mass spectrometry This review	R (Bioconductor), with Python and Java bridges	Modular ecosystem with shared data containers; literate, scripted workflows from raw mzML to annotated features	Analysts learning or building reproducible scripted pipelines
*mzmine* Schmid et al., 2023	Java desktop application with optional batch/CLI mode	End-to-end visual workflow from raw data to feature tables and molecular networks; strong GNPS integration	Analysts who prefer a GUI but want reproducible, exportable batch files
*MetaboAnalystR* Chong & Xia, 2018	R; web companion at MetaboAnalyst	Downstream statistical and pathway analysis on already-processed feature tables	Analysts whose entry point is a feature table, not raw data
*notame* Klåvus et al., 2020	R	Sensible default for normalisation, drift correction and feature selection for large clinical cohorts	Clinical metabolomics groups standardising QC and normalisation
*patRoon* Helmus et al., 2021	R	Unified wrapper around multiple non-target screening tools (*xcms*, OpenMS, MetFrag, SIRIUS, …); environmental and exposomics focus	Environmental, food-safety and exposomics researchers
*Workflow4Metabolomics/Galaxy* Giacomoni et al., 2015; Guitton et al., 2017	Galaxy web GUI, wraps R/Java tools	Hosted, GUI-driven pipelines on shared infrastructure; deployment and standardisation across institutions	Wet-lab analysts without programming; institutional core facilities

The comparison situates *Metabonaut* within the broader open-source landscape; it is not a feature-by-feature ranking. Tools differ in their design intentions and target audiences, and many are routinely used in combination

## Literate programming as the unifying medium

How an analysis is written down matters as much as the software it uses. We argue that literate analysis (i.e. prose, code and output woven into a single executable document) is the medium that makes a metabolomics analysis findable, reusable and teachable at once.

The idea has a long history. Donald Knuth introduced literate programming as an inversion of priorities: source should be written primarily for a human to read, with the machine instructions emerging from a coherent narrative (Knuth, 1992). Scientific computing later adapted this principle, first through Sweave and R Markdown and now through Quarto (Allaire et al., 2026), which lets an author interleave explanatory prose, executable code and its rendered output, such as tables, figures and console results, in one source file that compiles to a single document. We refer to this practice, applied to a complete data analysis, as literate analysis.

What this changes is the unit of scientific communication. In the conventional model, an untargeted metabolomics study is reported across separate artefacts: the methods section describes the analysis in prose; the figures and tables present its output; the supplementary materials hold the parameter values and ad-hoc steps; and, if the authors are diligent, a separate code repository holds the scripts. These can drift apart, and a reader has no reliable way to tell which is authoritative. A Quarto vignette collapses all four into one: because the document executes, the description of the analysis and the analysis itself are the same file. This is the concrete sense in which *Metabonaut*’s vignettes are FAIR analyses, not merely FAIR code.

Writing an analysis as plain text also makes it amenable to version control. Git can track a Quarto file line by line, so any two versions of an analysis can be compared directly (which parameter changed, which step was added), whereas a GUI session leaves no comparable record (Markowetz, 2015). Version control also turns an analysis into a citable object: a tagged release deposited in an archive such as Zenodo receives a DOI and can be retrieved unchanged.

Findability and version control do not, however, guarantee durability: a vignette degrades as R, Bioconductor and Python evolve, so reproducibility of the text is not the same as reproducibility of the result. Containerisation closes this gap: a container image is a self-contained snapshot of the whole computational stack, the operating system libraries, the R version, the pinned Bioconductor release and every package, sometimes also the data, the analysis depends on, frozen at publication time (Boettiger, 2015). For a user this replaces dependency management with two commands, installing a runtime such as Docker once and then pulling the archived image, and it is what allows a vignette to be re-executed years later in the same environment rather than merely read.

Text-based analysis has a further, practical advantage for learners. Because a script is plain text, a large language model can read it, explain it and suggest fixes: a learner who does not understand a peak-detection call can paste that code into an assistant and get an explanation, in whichever language they prefer. A sequence of menu clicks in a graphical tool offers no equivalent, as it leaves no text to share. Although this is not a claim about the future of artificial intelligence in research, that possibility further lowers the barrier to entry and encourages newcomers into the learning process.

## *Metabonaut*: design and pedagogy

### Origin, motivation and pedagogical principles

The motivation for *Metabonaut* is a gap in available training material. Individual package vignettes document individual tools, but few resources guide a newcomer through a complete untargeted LC-MS/MS analysis, from raw files to annotated and statistically assessed features, using a single coherent tool chain on a realistic dataset. Reviews of reproducible and open computational practice have repeatedly made the case that such end-to-end, executable examples are what let readers actually reuse an analysis rather than merely read about it (Grüning et al., 2018; Sandve et al., 2013). Metabonaut provides one such example, maintained openly with contributions from developers of the underlying Bioconductor packages.

Four principles shape the resource. First, it is *executable*,* not illustrative*: every code chunk in the vignettes can be executed, and the vignettes can be run together as one comprehensive analysis. Second, it is *versioned and citable*: each release is tagged on GitHub and archived on Zenodo under a concept DOI. Third, it is *containerised*: an archived Docker image containing the full software stack the vignettes depend on is provided, making long-term re-execution the default rather than a heroic exception. Fourth, it is *modular*: self-contained vignettes share serialised intermediate objects, so a learner can enter at any vignette without re-running the previous ones, and new vignettes describing alternative tools can be added without disturbing the existing ones. Three routes are available for running the vignettes: in the browser, as a Curated Workshop on the Galaxy-based Bioconductor Workshop Platform, which needs no installation at all; locally from the published container image, which reproduces every figure in this review without resolving dependencies by hand; or from a local R installation. All routes matter for teaching: a workshop or classroom can begin with every participant in an identical environment, rather than losing the first session to installation problems.

### The nine vignettes

**Fig. 2:**
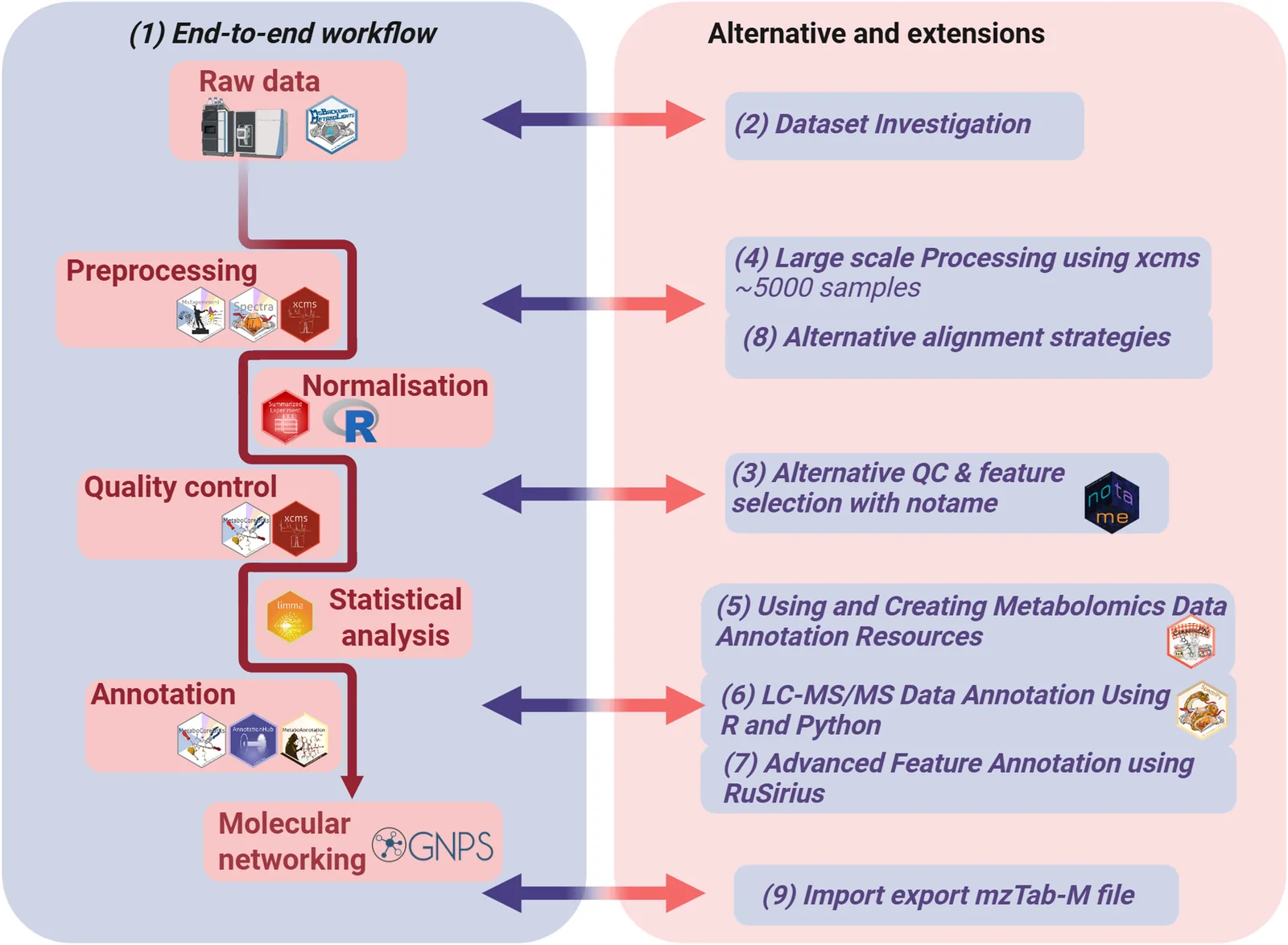
The Metabonaut vignettes. The detailed workflow (left) is the end-to-end vignette, carrying data from raw files to molecular networking; the other vignettes branch from it as alternatives or extensions, with package stickers shown where available

*Metabonaut* version 1.6.2 comprises nine vignettes. The keystone is the (1) *Complete End-to-End LC-MS/MS Metabolomic Data Analysis* vignette, which carries the *Metabonaut* plasma example (MTBLS8735) from preprocessing through statistical analysis to annotation, using (among many other packages) *xcms*, *MetaboAnnotation*, *SummarizedExperiment* (Martin Morgan, 2017) and *limma* (Ritchie et al., 2015). The other seven vignettes extend or offer alternatives to steps of this workflow. Three cover preprocessing: (2) *Dataset Investigation* (what to inspect before peak detection), (3) *Quality Control and Feature Selection Using notame*, and (4) *Large Scale Processing Using xcms* (4,063 samples on standard hardware, using the larger MTBLS93 cohort). Three cover annotation: (5) *Using and Creating Metabolomics Data Annotation Resources* (building libraries from GNPS and MassBank via *CompoundDb*), (6) *LC-MS/MS Data Annotation Using R and Python* (through *SpectriPy* and *matchms*), and (7) *Advanced Feature Annotation Using RuSirius* (SIRIUS formula assignment, structure-database search and de novo generation with MSNovelist). Finally, (8) *Seamless Alignment* merges new data with an already-processed reference dataset. Figure 2 shows how these vignettes relate to the end-to-end workflow. A ninth vignette, *mzTab-M file format: import-export of mzTab-M 2.1 files from SummarizedExperiment*, covers the standardised reporting format discussed in Sect. 7.1. While the code from all 9 vignettes can be executed, 2 contain also pre-computed output to allow automatic rendering through GitHub actions: vignette (4) *Large Scale Processing Using xcms* requires the download of a large experiment from MetaboLights (~ 600GB of MS data files) and has an overall execution time of over 17 h. Vignette (7) *Advanced Feature Annotation Using RuSirius* needs a registered SIRIUS user account. All other vignettes execute in less than 10 min and can be evaluated interactively (see Table 3 for timing, disk, and memory usages).

**Table 3:** Code execution overview for the individual vignettes

Vignette no.	Data	Internet	Runtime	Memory	Disk space
(1)	yes	yes	00:04:40	2.4	1.3
(2)	yes	yes	00:03:10	2.2	1.0
(3)	no	no	00:00:40	1.2	0.0
(4) ^✳^	yes	yes	17:30:00	8.6	611
(5)	yes	yes	00:05:30	4.2	0.9
(6)	no	no	00:00:20	0.95	0.0
(7) ^✳ †^	yes	yes	00:00:50	0.97	0.0
(8)	yes	yes	00:02:20	1.6	1.5
(9)	no	no	00:00:20	1.1	0.0

^✳^ vignettes with pre-computed content. ^†^ vignette requiring an external account. *Data*: whether data needs to be retrieved from an external repository (MetaboLights, Zenodo, Bioconductor). *Internet*: whether an active internet connection is required. *Runtime*: approximate execution time [hh: mm: ss]. *Memory*: approximate peak memory used by the code [GB]. *Disk space*: approximate amount of additional local disc space required to run the vignette [GB]

### Availability, licensing and contribution

The source code of Metabonaut is available in the GitHub repository https://github.com/RforMassSpectrometry/Metabonaut. Metabonaut’s Docker image is provided through docker hub at https://hub.docker.com/r/rformassspectrometry/metabonaut. Releases are tracked and archived on Zenodo under the concept DOI https://doi.org/10.5281/zenodo.15062929. This manuscript describes Metabonaut version 1.6.2 (Docker image metabonaut: v1.6.2, index digest sha256:e17ab1dd7b397798d70c1bf03c923dfa4ea6f7b96263d79d92eb748f47e38ef2). Pre-rendered analysis report documents can be browsed at the project’s GitHub pages site https://rformassspectrometry.github.io/Metabonaut. As of July 2026 an interactive, cloud-based instance of Metabonaut is available as a Curated Workshop on the Bioconductor Workshop Platform (https://workshop.bioconductor.org/*)* so the individual vignettes can be executed and tested without any local software installation.

*Metabonaut* is openly developed through GitHub pull requests, its content released under CC BY-SA 4.0. Several vignettes were written by people other than the *Metabonaut* maintainers: the *notame* (Klåvus et al., 2020) vignette was contributed by an author outside the core team, and the *SpectriPy* (Graeve et al., 2025) vignette by that package’s own developers within the R for Mass Spectrometry project. Contribution is not limited to writing vignettes. Others have added infrastructure, such as the Galaxy deployment, or reviewed the end-to-end workflow for the readers it is aimed at: researchers with wet-lab rather than computational backgrounds, whose advice shaped how much is explained and where a newcomer is likely to get stuck. That mix mirrors the range of backgrounds among the authors of this review. This open model is itself part of the pedagogy, demonstrating that the resource is maintained the way a reproducible data analysis should be.

## Visual inspection and quality control

A common objection to script-based analysis is that it sacrifices the visual intuition that graphical tools provide. The opposite is closer to the truth: because plots are generated from code, they can be reproduced, parameterised and applied uniformly across an entire dataset. This section illustrates the point through two *Metabonaut* vignettes (*Dataset Investigation* and *Quality Control and Feature Selection*) and a more general argument about inspection at scale.

The *Dataset Investigation* vignette walks learners through what to examine before peak detection: base-peak chromatograms by sample group, total ion chromatogram across injection order, background signal by study group, and mass-accuracy diagnostics. Its framing is deliberately practical, encouraging the analyst to think critically about the data and to flag or remove problematic samples early, rather than discovering hours into an analysis that some should have been excluded. None of these diagnostics is novel in itself; the contribution is to show them as reproducible, scripted steps that travel with the analysis.

**Fig. 3:**
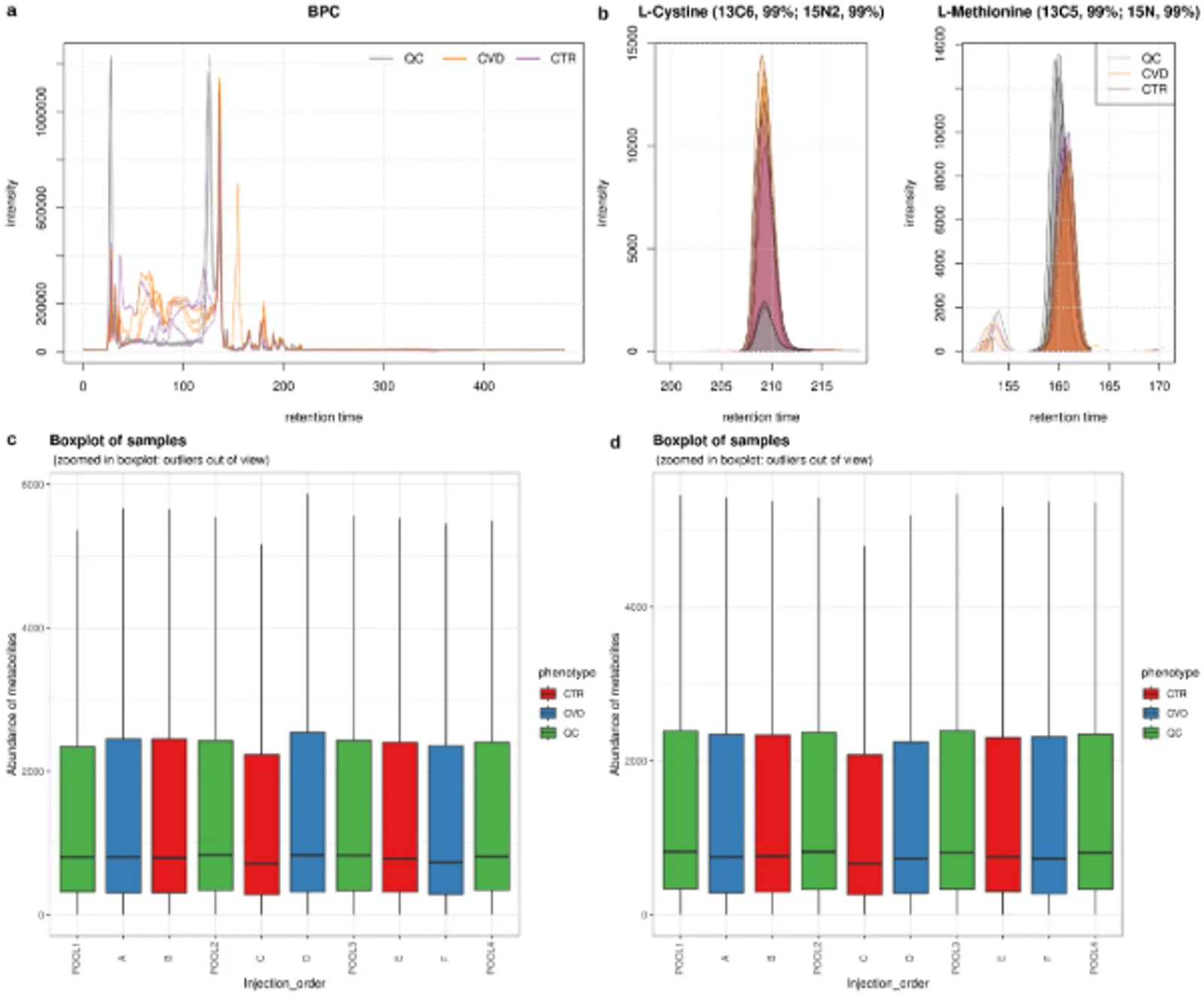
Visual inspection and quality control of the **Metabonaut** plasma example (MetaboLights MTBLS8735). **a** Base-peak chromatograms of all study samples, coloured by sample group (QC, pooled quality-control samples; CVD, cardiovascular-disease cases; CTR healthy control). **b** Extracted-ion chromatograms of two isotope-labelled internal standards, L-Cystine (13C6, 99%; 15N2, 99%) and L-Methionine (13C5, 99%; 15 N, 99%), across the same samples. **c**, **d** Distribution of feature abundances per sample, ordered by injection sequence and coloured by phenotype, at two stages of the quality-control workflow. All panels are produced by code within the vignettes and can be regenerated for any dataset

The *Quality Control and Feature Selection* vignette demonstrates quality-control-driven normalisation and feature selection with *notame* (Klåvus et al., 2020), which handles drift correction, batch effects, QC-based filtering and missing-value imputation. The Bioconductor refactor of *notame* onto the *SummarizedExperiment* data model lets preprocessing flow directly into quality control, normalisation and feature selection, with no data hand-off between steps. The vignette implements established community standards for QC samples (Broadhurst et al., 2018; Kirwan et al., 2022) and uses random-forest imputation (Kokla et al., 2019), so a learner adopts field conventions rather than ad-hoc choices. The vignette is explicit about the consequences of the imperfect QC design noted in Sect. 1.4: the differing QC source may make the dilution-factor estimate inaccurate and drift correction is likely to suffer, and in one assessment the QC samples appear more dissimilar after normalisation, which can indicate that unwanted variation has increased. Figure 3 shows representative diagnostics produced by these two vignettes, from raw-signal inspection through to the per-sample abundance distributions used to judge quality.

Scripted plotting also scales in a way that manual inspection does not. The same plotting code can be applied to every sample in a dataset: a single command produces a grid of chromatograms across hundreds of samples, or the extracted-ion chromatogram of one feature across an entire cohort, faceted by injection order or study group. Manually opening and inspecting each sample in a graphical tool does not scale to large studies, whereas a scripted plot is written once and reused unchanged, whatever the number of samples.

## Reusing public data and scaling to large cohorts

Public data can be downloaded and reused from graphical tools as well as from scripts. What a scripted, programmatic connection to an archive adds is that the retrieval itself becomes part of the reproducible analysis: the dataset is identified by its accession in code, fetched the same way on every run, and processed without manual download or file handling. This makes reuse *reproducible*, not merely possible. Once an archive entry can be loaded directly into an analysis, scaling to large cohorts becomes the natural operational consequence of taking reuse seriously. This section develops that argument through three vignettes (*Using and Creating Metabolomics Data Annotation Resources*, *Seamless Alignment* and *Large Scale Processing*).

The public metabolomics archives, MetaboLights (Yurekten et al., 2024), Metabolomics Workbench (Sud et al., 2016), GNPS/MassIVE (Wang et al., 2016) and MassBank (Horai et al., 2010) now hold thousands of studies between them, yet a deposited dataset is typically treated as an endpoint. The *MsBackendMetaboLights* backend package changes this by turning an archive identifier into a *Spectra* object directly, without manual download or file-path handling. A deposited dataset can therefore serve as a direct input to a new analysis, not only as an endpoint of the study that produced it. The vignettes demonstrate this with *MsBackendMetaboLights* for MetaboLights, but the same mechanism applies to the other archives through their respective backends (for example *MsBackendMassbank* and *MsBackendMassIVE*), which expose the same interface.

The *Annotation Resources* vignette walks through pulling public spectra from GNPS and MassBank and packaging them as a versioned *CompoundDb* resource. Also the selection of the annotation resource, subset or version are analytical parameters that are important to report. The benefit of the described approach is the full traceability of employed data sources: the resulting library ships with the analysis, so a downstream user reproduces not only the feature table but the identifications behind it. Library sharing is the under-developed counterpart to data sharing, solved infrastructurally for raw spectra through MassBank and GNPS, but not yet for the derived, project-specific reference libraries on which most annotation depends. The vignette shows how to retrieve reference spectra for the compound classes relevant to a given study, filter them by quality and metadata, and assemble them into a single self-contained *CompoundDb* database. Packaging that selection, which is rarely reported in enough detail to reproduce, as a versioned resource makes it explicit and reusable: another group can apply the same library to their own data, or extend it, and obtain comparable identifications.

**Fig. 4:**
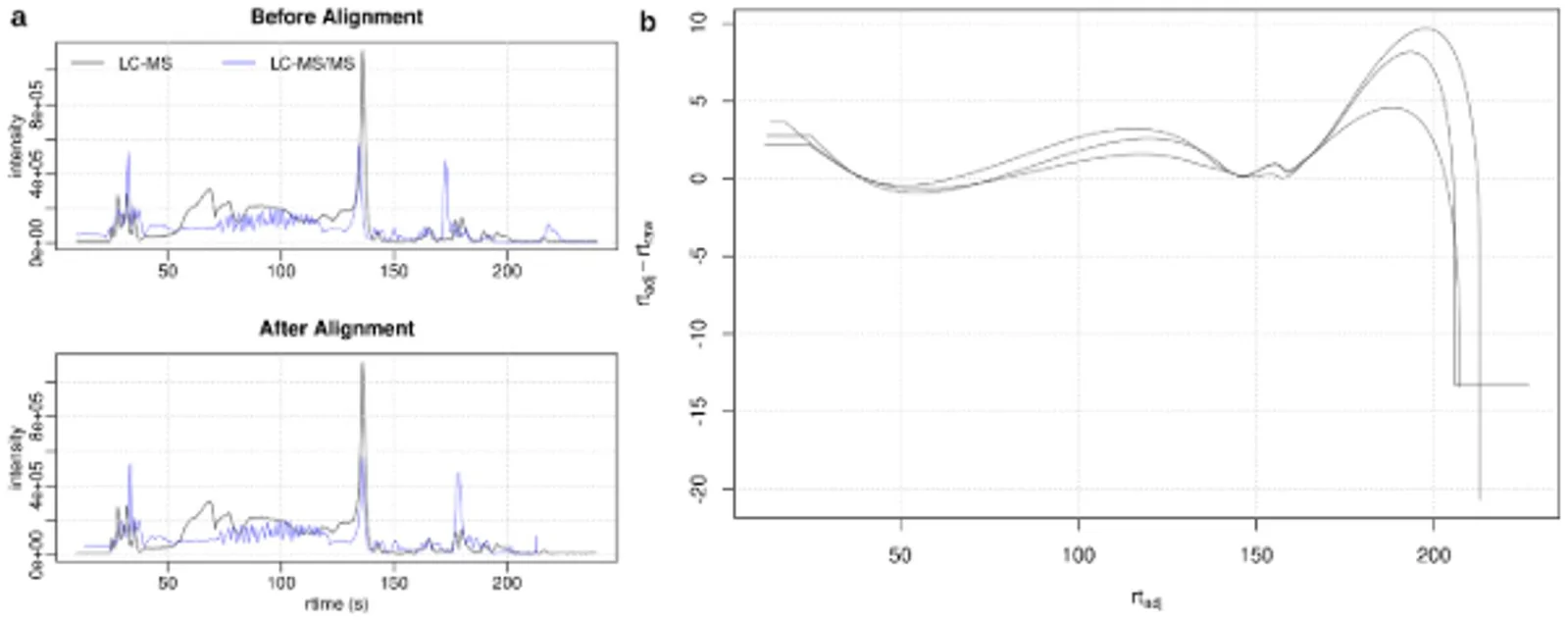
Seamless alignment of a newly measured dataset onto an already preprocessed reference dataset. **a** Base-peak chromatograms of an LC-MS and an LC-MS/MS measurement before (top) and after (bottom) retention-time alignment. **b** Retention-time adjustment applied to each sample, shown as the difference between adjusted and raw retention time as a function of adjusted retention time

The *Seamless Alignment* vignette aligns features, in mass-to-charge and retention-time space, between a learner’s new cohort and a previously preprocessed reference. Because that reference can be a public MetaboLights study loaded directly through *MsBackendMetaboLights*, public data becomes a comparison object rather than only a deposition target. This supports cross-study comparison without joint reprocessing, longitudinal expansion of a growing cohort, and transfer of identifications from a well-characterised study to a new one. Figure 4 illustrates the alignment step and the retention-time adjustment it applies.

The *Large Scale Processing* vignette demonstrates this on a MetaboLights study of 4,063 samples, using on-disk *Spectra* backends to enable processing also on standard compute infrastructure. The decisive point for a learner is continuity: the *xcms* idioms used on a dozen files in the end-to-end vignette are *identical* on thousands of files here; only the storage backend changes. In a script-based world, scaling is a configuration change, not a workflow rewrite.

Together, these capabilities demonstrate several forms of public data reuse. Deposited studies can be reanalysed with updated methods, and the reanalysis itself deposited as a citable report alongside the original data. Cohorts can be compared in feature space without joint reprocessing. Aligning this practice with established metabolomics reporting standards (Spicer et al., 2017; Sumner et al., 2007) would make deposited reanalyses easy to find and to build on.

## Interoperability to extend analytical capacity

No single language or ecosystem holds the best tool for every step of an untargeted metabolomics workflow. *Metabonaut* therefore shows how to combine tools from different languages within one analysis, demonstrating two bridges that let an analysis reach beyond R without leaving the literate document.

*SpectriPy* (De Graeve et al., 2025) wraps Python’s *matchms* (Huber et al., 2020) so that R workflows can call Python-native algorithms in the same Quarto document. The *R and Python annotation* vignette filters and scores MS2 spectra with the ModifiedCosine algorithm in *matchms*, then brings the results back into R for interpretation. This lets the analyst use the most suitable algorithm for a given step regardless of the language it is implemented in.

*RuSirius* gives programmatic access to SIRIUS (Böcker et al., 2009; Dührkop et al., 2019), so the same document can drive SIRIUS formula assignment, CSI: FingerID and COSMIC structure ranking (Hoffmann et al., 2022) and MSNovelist *de novo* structure generation (Stravs et al., 2022). The *Advanced Feature Annotation* vignette teaches a clear annotation logic: library search first, COSMIC for orthogonal confirmation or for features without a database match, and MSNovelist for genuinely novel structures. *De novo* methods complement rather than replace library search, and the vignette is explicit about which tool answers which question.

### Standardised output: making results FAIR

Interoperability also concerns the results a workflow produces, not only the tools it calls. mzTab-M (Hoffmann et al., 2019) is the HUPO-PSI standard reporting format for metabolomics, designed so that a quantitative result and the evidence behind it travel together, which a generic table export does not preserve. A dedicated vignette demonstrates import and export of a completed analysis to an mzTab-M file using the *RmzTabM* package. Writing results in a defined, tool-agnostic format has practical value: the feature table, its identifications and their supporting evidence become readable by other software without bespoke parsing, can be deposited alongside the raw data, and remain interpretable independently of the R session that produced them. The vignette teaches the export step by step, showing how a completed analysis is written to an mzTab-M file and walking through the resulting file section by section, so that the export is understood rather than treated as a black box. By promoting standardization, the vignette eliminates the undocumented manual steps where analyses typically lose reproducibility. Ultimately, it extends FAIR principles to the results themselves: reported in a documented, community-standard format based on controlled vocabularies, the findings become interoperable and reusable, and not only the analysis that produced them.

## Discussion and conclusion

*Metabonaut* is a teaching resource, not a comprehensive software suite: it presents one documented path through a set of packages rather than documenting every available tool. Two boundaries follow. R remains a genuine learning investment; and supervisors should budget for it. In addition, the resource covers LC-MS and LC-MS/MS only: NMR and ion-mobility MS are out of scope for the current version. For the latter, native four-dimensional feature detection is not yet available in the R-based stack and a centWave-equivalent for LC-IMS-MS would be a valuable addition, though such data can already be preprocessed and exported with mzmine and the resulting feature tables imported into R for the downstream steps described here, and support in the underlying *Spectra* package continues to develop. Nevertheless, the underlying logic of this pipeline can be adapted to process data from other MS-hyphenated separation techniques, such as gas chromatography (GC) and capillary electrophoresis (CE).

Similar resources to Metabonaut might be developed in future covering also other aspects of metabolomics data analysis. However, development of such educational resources involves substantial long-term commitment, and funding, something difficult to find with current funding schemas and a focus on the development of new tools instead of improving documentation. In general, such workshops should be tailored for data analysts and should, similarly to Metabonaut, and following the principle of literate programming, provide not only the full source code to perform an analysis but also explanatory text on methods and parameter choices. To lower the accessibility and installation hurdle, we would advise the teaching resource to be accessible also as a cloud service, which would however require sustained long-term funding.

The bottleneck in untargeted metabolomics is no longer the availability of tools; open-source software now covers essentially every step from raw data to annotation. The bottleneck we address is *training*. Most researchers entering the field are taught graphical tools first and adopt script-based analysis only later. Training resources such as *Metabonaut*, the Carpentries and the Galaxy Training Network (Batut et al., 2018), together with their equivalents in other ecosystems, help close this gap. Depositing raw data is necessary but not sufficient for reproducibility: without the analysis that produced the reported feature table, a deposited dataset cannot be recomputed. Script-based, container-pinned, literate workflows make that data analysis available in a form that others can inspect and re-execute.

### Outlook

Several developments would extend *Metabonaut*’s reach. Community-curated, openly shared *CompoundDb* reference libraries, analogous to the shared raw-spectrum resources provided by MassBank, would increase the value of the annotation-resource vignette. Closer ties between this reuse infrastructure and the deposition standards of MetaboLights and Metabolomics Workbench, including reanalysis examples deposited alongside the originals, would be a natural next step. We would consider this review a success if it encouraged comparable resources in other ecosystems and if reviewers came to expect, as default practice, that a metabolomics paper’s analysis be deposited as a citable, re-executable artefact. Much of the progress on the data side of FAIR followed from obligation rather than goodwill: journals and funders now routinely require raw data deposition as a condition of publication or of grant reporting, and compliance rose accordingly. We expect the analysis side to follow the same path, and consider it a reasonable goal that depositing a re-executable analysis becomes an expectation of publishing rather than an act of individual diligence.

## Data Availability

The datasets discussed in this review are publicly available in MetaboLights under accessions MTBLS8735 and MTBLS93.

## Code availability

The source code of Metabonaut is available at https://github.com/RforMassSpectrometry/Metabonaut. Tagged releases are archived on Zenodo under the concept DOI https://doi.org/10.5281/zenodo.22300919, the rendered vignettes are available at https://rformassspectrometry.github.io/Metabonaut, and the Docker image is available at https://hub.docker.com/r/rformassspectrometry/metabonaut.
